# GAMEC – a new intensive protocol for untreated poor prognosis and relapsed or refractory germ cell tumours

**DOI:** 10.1038/sj.bjc.6603865

**Published:** 2007-07-03

**Authors:** J Shamash, T Powles, W Ansell, J Stebbing, K Mutsvangwa, P Wilson, S Asterling, S Liu, P Wyatt, S P Joel, R T D Oliver

**Affiliations:** 1 Department of Medical Oncology, St Bartholomew's Hospital, London, UK

**Keywords:** GAMEC, methotrexate, high dose, germ cell

## Abstract

There is no consensus as to the management of untreated poor prognosis or relapsed/refractory germ cell tumours. We have studied an intensive cisplatin-based regimen that incorporates high-dose methotrexate (HD MTX) and actinomycin-D and etoposide every 14 days (GAMEC). Sixty-two patients were enrolled in a phase 2 study including 27 who were untreated (IGCCCG, poor prognosis) and 35 with progression despite conventional platinum based chemotherapy. The pharmacokinetics of the drugs were correlated with standard outcome measures. Twenty of the untreated patients were progression free following GAMEC and appropriate surgery, as were 18 individuals in the pretreated group. None of the established prognostic factors for therapy for pretreated patients could identify a poor-prognosis group. Five out of nine late relapses to prior chemotherapy were progression free following GAMEC and appropriate surgery. All patients had at least one episode of febrile neutropenia and there were five (8%) treatment-related deaths. PK values were not predictive of efficacy or toxicity, although the dose intensity in the pretreated group of patients, especially of HD MTX, was significantly correlated with progression-free survival (PFS). GAMEC is a novel intensive regimen for this group of patients producing encouraging responses, although with significant toxicity. For those in whom it fails, further therapy is still possible with durable responses being seen.

The treatment of germ cell tumours (GCT) is one of the major successes of cytotoxic chemotherapy, with the three-drug BEP regimen (bleomycin, etoposide and cisplatin) being most established. Four cycles of BEP are considered optimal for those with untreated metastatic disease with the poorest prognosis in the routinely used IGCCCG classification (International Germ Cell Collaborative Group, 1997). For these individuals, the cure rate is approximately 50%, and to date, no regimen in any randomised controlled trial has shown a superior outcome (Kaye *et al*, 1998; Hinton *et al*, 2003), although higher cure rates have been seen in various phase II studies (Horwich *et al*, 2006).

Patients who relapse after first-line therapy have a durable cure rate of between 25–60%, with the most significant prognostic factors being the site of the primary, the response to initial therapy, the duration of this response and the level of serum tumour marker at relapse (McCaffrey *et al*, 1997). Cisplatin, ifosfamide and a third drug (paclitaxel, etoposide or vinblastine) are used most commonly with or without high-dose chemotherapy consolidation (Rick *et al*, 2001; Kondagunta *et al*, 2005; Mead *et al*, 2005). Others have used a tandem or triple stem cell transplant after initial stem cell mobilisation (Bhatia *et al*, 2000; Motzer *et al*, 2000).

On the basis of encouraging phase 2 data, several more complex regimens have been utilised for untreated patients, and these have generally intensified the drug cisplatin using a dose-dense approach (including BOP/BEP (Anthoney *et al*, 2004), CBOP/BEP (Christian *et al*, 2003)) or an approach in which additional agents are added (such as POMB/ACE; Bower *et al*, 1997). In certain patients, an etoposide dose of 500 mg m^−2^ is superior to 360 mg m^−2^ (Toner *et al*, 2001) (its absence from the initial few weeks of the BOP/VIP-B regimen may account for the failure to show an advantage of that treatment over BEP (bleomycin, etoposide and cisplatin); Kaye *et al*, 1998).

In relapsed disease, we have found that a dose-dense cisplatin using m-BOP (weekly cisplatin, MTX, bleomycin and vincristine), led to long-term remissions in 42% of patients (Shamash *et al*, 1999). In addition, the combination of etoposide, actinomycin-D and MTX (EAM) without cisplatin produced a complete remission (CR) rate of 21% in patients who had relapsed after PVB (Levi *et al*, 1990). This led us to develop granulocyte colony stimulating factor, actinomycin-D, methotrexate, etoposide, cisplatin (GAMEC), which was designed to achieve dose-dense delivery of cisplatin, early introduction of etoposide in a similar dose to conventional BEP, the incorporation of sliding scale MTX at high dose and add actinomycin-D to the therapy, with appropriate colony factor support.

Pharmacokinetics (PK) relationships for toxicity and efficacy endpoints have been documented for high-dose methotrexate (HD MTX) in osteosarcoma (Crews *et al*, 2004) and acute lymphoblastic leukaemia (ALL), and for teniposide in ALL (Evans *et al*, 1998). As this is a novel regimen, PK studies on MTX, 7-hydroxy MTX (its metabolite implicated in renal dysfunction), etoposide and actinomycin-D were conducted to identify possible relationships between PK parameters and either toxicity or efficacy. We also investigated the overall survival, time to progression and toxicities using this regimen.

## PATIENTS AND METHODS

St Bartholomew's Hospital, London is one of the largest referral centres for GCT in the United Kingdom. Between September 1997 and June 2005, 62 patients were recruited for a phase 2 trial of GAMEC chemotherapy, with appropriate ethical approval and written informed consent. Twenty-seven were untreated patients with poor prognosis disease using the IGCCCG criteria and 35 had relapsed following at least one line of conventional platinum-based therapy.

**Figure 1:**
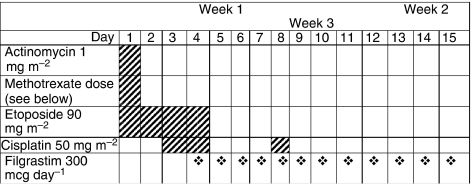
GAMEC chemotherapy schedule. Week 1: actinomycin-D (1 mg m^−2^) day 1: etoposide: 90 mg m^−2^, days 1–4 over 2 h; MTX loading dose over 30 min followed by 12 h infusion; day starting 4 h after the etoposide: cisplatin 50 mg m^−2^ over 4 h days 3 and 4. MTX was given as follows, depending on GFR: >120 ml min^−1^, 2 g m^−2^ loading then 8 g m^−2^ over 12 h (use 6g m^−2^ over 12 h if >30 years or PS>1); 100–119 ml min^−1^, 2 g m^−2^ loading, then 6 g m^−2^ over 12 h; 80–99 ml min^−1^, 2 g m^−2^ loading, then 4 g m^−2^ over 12 h; 60–79 ml min^−1^, 2 g m^−2^ loading, then 3 g m^−2^ over 12 h; 40–59 ml min^−1^, 1.5 g m^−2^ loading followed by 2 g m^−2^ over 12 h; 20–39 ml min^−1^, 1 g m^−2^ loading only. Acetazolamide was prescribed 500 mg 2 × per day for 3 days. Regular sodium bicarbonate (100 mmol), 6 h with 20 mmol KCl in 5% glucose for 48 h. Folinic acid was started 30 h post-MTX treatment. Folinic acid: rescue commenced at 30 h post-MTX treatment. The first level of MTX was taken 24 h post-infusion start. Initial rescue was 100 mg i.v. over 30 min, followed by 250 mg over 24 h. If the 24-h level was <2 *μ*mol l^−1^ continue with folinic acid 30 mg, six hourly for 3 days, otherwise give a further infusion of 350 mg over 24 h and recheck level at 48 h. If the 24 h level >40*μ*mol l^−1^ the folinic acid was increased to 700 mg per 24 h. At 48 h and beyond: if the level were >3 *μ* l^−1^ in the presence of renal impairment, the use of carboxypeptidase was considered. If renal function was preserved, an infusion of 700 mg per 24 h was continued; if the level was >1 and <3*μ*mol l^−1^, 350 mg was given over 24 h, and the level was checked every 24 h. When the level was <1, the dose was reduced to 30 mg orally 4 × per day, until the level was <0.2 *μ* l^−1^. Haematological parameters: filgrastim (300 mcg per day) was started on day 4 and continued until WBC>3. To start each cycle, it was necessary to have neutrophils>1 × 10^9^ l^−1^ and platelets>60 × 10^9^ l^−1^. Renal parameters: U and E and creatinine (Cr) were measured daily, and if Cr rose >20%, no cisplatin was given until Cr reduced below that level. If the serum creatinine rose clearance by 15%, an EDTA clearance was repeated. If the clearance was <40 ml min^−1^, carboplatin AUC 4 was substituted for cisplatin. In this case, vincristine was given on weeks 2, 4, 7 and 9. If the clearance subsequently improved, then cisplatin was reintroduced and vincristine was dropped. Dose reductions: a 20% dose reduction was made in the doses of cisplatin, actinomycin-D and etoposide, in the presence of platelets<20 × 10^9^ l^−1^ or neutrophils<0.5 × 10^9^ l^−1^ for>5 days. A 20% reduction in the dose of MTX was made in the presence of grade 3 or 4 mucositis. A 50% dose reduction in the dose of MTX was made, if the 24-h level was >80 *μ* l^−1^ or the 48 h level was >4 *μ* l^−1^.

Relapse was defined as the appearance of new disease or the development of increasing tumour markers in patients with known sites of disease. Patients receiving first-line therapy whose markers although not declining at the rate predicted by their known half-lives, were nevertheless still falling, were ineligible. Such patients completed their current therapy and only became eligible if their markers started to rise. Histological diagnosis was not mandatory in untreated patients who had a testicular mass and elevated tumour markers, if it were felt that delay of chemotherapy would compromise survival; all patients were staged using whole-body CT before therapy and all had renal function assessed using EDTA glomerular filtration rate (GFR) clearances. The GAMEC schedule is shown in Figure 1.

An induction cycle of baby BOP (cisplatin 50 mg m^−2^, vincristine 2 mg and bleomycin 30 000 U over 12 h) was given to patients who had renal obstruction, respiratory failure due to disease, extensive inferior vena cava thrombosis thought to be at high risk of pulmonary embolism and patients with poor performance status (Eastern Co-operative Oncology Group PS 3). In these individuals, GAMEC was given 10–14 days later with omission of cycle 1 day 8 cisplatin. Drug doses were calculated on the basis of body surface area; the full dose was deemed to be 100%, if a 20% dose reduction was needed then the dose was said to be 80%. This percentage dose was multiplied by the dose delivery (actual inter-cycle length divided by intended inter-cycle length expressed as a percentage). The drugs were analysed separately and then combined, assuming the contribution of each drug to be equal.

At the end of therapy, patients underwent surgery to remove sites of disease greater than 1 cm in diameter (3 cm in seminoma). If any viable germ cell tumour elements were found, no further therapy was given, unless the patient relapsed. In patients with brain metastases, radiotherapy was not routinely administered. Patients who progressed subsequently were offered further chemotherapy with high-dose chemotherapy consolidation.

Radiological assessment was carried out before week 6, unless tumour markers showed a response. Treatment was not stopped if tumour markers failed to decline at anticipated half-life. Tumour markers including alpha foetoprotein (AFP) and human chorionic gonadotrophin (*β*HCG) were routinely followed. In some patients, the AFP rose during therapy, while the patient was responding due to AFP production by the liver and additional scanning was used to clarify this situation.

### Pharmacokinetics

Twenty-four EDTA blood samples were collected over a 72 h time period from the start of treatment. Plasma was separated by centrifugation (1200 **g** for 10 min) and stored at −40°C until analysis. Methotrexate, 7-hydroxymethotrexate (7-OH MTX) and etoposide were detected by two separate HPLC methods with UV detection. Actinomycin-D was measured by a novel LC-MS-MS method with a sensitivity of <1 ng ml^−1^ (Veal *et al*, 2005). PK analysis was carried out in Kinetica (Innaphase Corp, Philadelphia, PA, USA) using non-compartmental methods. Area under the concentration–time curve (AUC) was determined by the trapezoidal method, using the linear rule for ascending concentrations and the log linear rule for descending concentrations. Area under the concentration–time curve was extrapolated to infinity by dividing the concentration at 24 h by the elimination rate constant (*λ*_z_), to derive AUC_0–*∞*_. Total plasma clearance was derived from the dose divided by AUC_(0−*∞*)_, or from steady-state concentration at 12 h (C_SS_) divided by the infusion rate (mg h^−1^). Elimination half-life (*t*1/2) was calculated as 0.693/*λ*_z_.

### Statistical methods

Statistical analyses were performed with the STATA 8.2 statistical software package. Overall survival (OS) and PFS were assessed using the Cox proportional hazard method for continuous variables. Survival curves were generated using the Kaplan–Meier method. Categorical variables were analysed using the log-rank test. To assess the correlation between the various categorical variables and being progression free (PF) to GAMEC, Fisher's exact test was used. The median test was used to examine continuous variables against PF status. Logistic regression was used to determine which factors were predictive of being PF. PK between group comparisons were made using the Mann–Whitney *U*-test. Progression-free and overall survivals were analysed on an intention to treat basis.

## RESULTS

**Figure 2:**
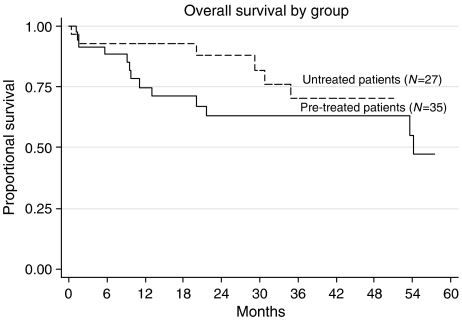
Overall survival.

The majority of patients had non-seminomatous GCT and the median age measured was 33 years (Table 1). At a median follow-up of 2.5 years, 20 (74%) untreated patients were PF following GAMEC and appropriate surgery (Table 2; Figure 2). There were two treatment-related deaths (TRDs), one additional patient was salvaged by further therapy (78% overall). Out of five patients with central nervous system metastases at presentation, three remain PF.

Eighteen (51%) of the pretreated group were similarly PF and a further four were salvaged by additional therapy (63% overall). There were three TRDs. Nine pretreated patients had late relapses before GAMEC and five remain PF. Three patients had brain metastases on relapse, two had post-chemotherapy surgery confirming CR and that they were PF and one progressed and died. Eight patients had viable cancer at surgery, five of them had greater than 10% viable tumour and all five relapsed. The remaining three with less than 10% viable tumour were all PF.

High-dose chemotherapy was offered to 10 patients. In the untreated group, four received high-dose therapy, but it was unsuccessful in all of them. One received high-dose carboplatin AUC20 with etoposide 1600 mg m^−2^ and cyclophosphamide 6 g m^−2^ Another patient received high-dose carboplatin AUC20 with thiotepa 500 mg m^−2^ and cyclophosphamide 6 g m^−2^. A third patient received carboplatin AUC10 with irinotecan 200 mg m^−2^ and gemcitabine 360 mg m^−2^ over 72 h, supported by autologous stem cells and repeated and given sequentially three times. The fourth patient received high-dose carboplatin AUC20 with etoposide 1600 mg m^−2^ and melphalan 140 mg m^−2^. In the pretreated group, high-dose therapy consisted of high-dose carboplatin AUC20 with etoposide 1600 mg m^−2^ and cyclophosphamide 6 g m^−2^ in two patients and was successful in one. High-dose carboplatin AUC30 and etoposide 1600 mg m^−2^ was offered to one patient, it but resulted in a TRD. High-dose topotecan 30 mg m^−2^, carboplatin AUC21 and thiotepa 500 mg m^−2^ was offered to three patients as part of a clinical trial, and it was successful in two.

### Toxicity

The most common toxicities were myelosuppression and mucositis (Table 3a). There were five TRDs (8%); four of these were due to sepsis and one was due to intra-abdominal haemorrhage from choriocarcinoma. A large number of cycles were complicated by febrile neutropenia, with about half of them requiring platelet transfusion (Table 3b).

Significant reversible renal dysfunction occurred in five of the untreated patients (grade 2 WHO criteria), of whom one was dialysed for 24 h following an episode of septic shock. His renal function before this had been normal and returned subsequently to normal. In the pretreated group, four were similarly affected, one of whom (who had significant renal problems with BEP) became dialysis dependent long term. A total of 6% of cycles required the omission of cisplatin, and in one-third of these cycles carboplatin was substituted. One patient required carboxypeptidase to inactivate MTX during the first cycle of treatment; his EDTA clearance before HD MTX had been normal and he did not receive any cisplatin on that cycle. Methotrexate was cautiously reintroduced on the third cycle without adverse effects after his renal impairment had subsided.

Two patients developed typhlitis, one required a defunctioning colosostomy that was subsequently reversed. Three patients developed thrombo-embolic disease (all central access line associated). Two patients required parenteral feeding. One patient developed transient occipital blindness, he had received two lines of prior cisplatin-based therapy. An MRI scan confirmed white-matter changes in the occipital lobe, thought to be due to cisplatin and not metastases. His vision subsequently recovered without further therapy.

### Prognostic factors

**Figure 3:**
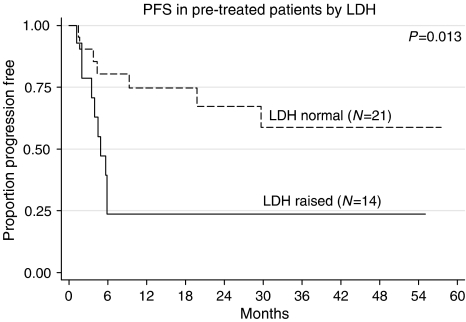
PFS in pretreated patients by absence or presence of raised LDH.

**Figure 4:**
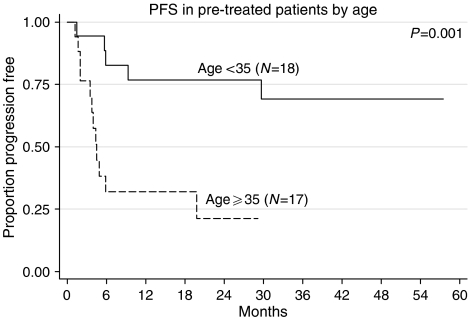
PFS in pretreated patients by age (above and below median).

**Figure 5:**
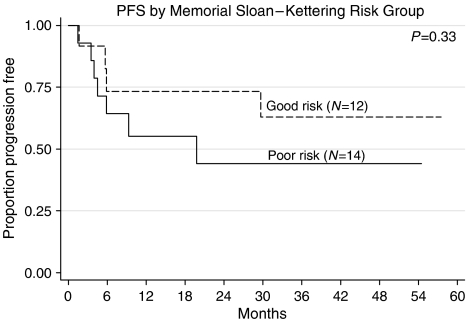
PFS by Memorial Sloan-Kettering Risk Group.

We looked at the following prognostic factors to see if they correlated with PF status. They were age, presence of non-seminomatous histology, raised lactate dehydrogenase (LDH), Memorial Sloan-Kettering Cancer Center (MSKCC) criteria and Medical Research Council (MRC) UK criteria for relapsed GCT outcome. Only age (<median age *vs* >median age: 72 *vs* 29%, FET (Fisher's exact test)=0.018) and raised LDH before GAMEC (normal LDH *vs* raised LDH: 67 *vs* 29%, FET=0.041) were significant. In the pretreated group, neither the MRC criteria for adverse outcome (*β*HCG or *α*FP >100, failure to achieve a CR to initial chemotherapy, relapse within 2 years) (Fossa *et al*, 1999) nor those developed by MSKCC (Motzer *et al*, 2000), including extragonadal primary, failure to achieve at least marker-negative partial response (m−ve PR), failure of two lines of cisplatin-based therapy), were able to define a poor-prognosis group. The good-risk group defined by the MSKCC (gonadal primary, one line of cisplatin-based therapy, achievement of at least m−vePR) (7/14 PF) and relapse at least 6 months from the end of the last chemotherapy, also failed to define a group with a better prognosis (8/12 PF). Only age and raised LDH were significant on multivariate analysis (Figures 3, 4 and 5).

### Dose delivery and outcome

**Figure 6:**
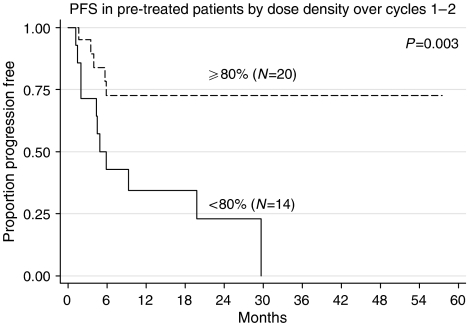
PFS in pretreated patients by dose density over cycles 1 and 2.

In the untreated group, no relationship between dose delivered or dose density was seen. In the pretreated group, when the two components of dose density were entered as separate variables into a regression model, both dose and inter-cycle delay were found to be independently associated with PFS (Figure 6). The ideal (100% dose) for MTX was adjusted for renal function, as shown in Figure 1. The number of patients maintaining the dose of MTX at ⩾80% over the first 2 cycles was also significant; no other drug alone showed a significant effect. However, for the four drugs together, the overall dose density delivered was highly significant (Table 4). Beyond the first two cycles, the effect of dose density was no longer statistically significant for PFS.

### Pharmacokinetics and pharmacodynamics

Pharmacokinetic data was obtained on 43 patients for MTX and etoposide, and 31 patients for actinomycin-D. Summary data are presented in Table 5. There was no relationship between renal function (serum creatinine) and plasma clearance of MTX, actinomycin-D or etoposide (*r*^2^<0.1), although only seven patients had serum creatinine values above the normal range (>115 *μ*mol l^−1^), and all were <139 *μ*mol l^−1^.

The main aim of the pharmacological aspect of the study was to identify relationships between pharmacokinetic parameters and pharmacodynamic effects. The primary pharmacodynamic measures were PF status for efficacy, and neutrophils <0.5 × 10^9^/l, for <4days or ⩾4 days for toxicity. AUC_0−*∞*_ was taken as the primary PK measure. methotrexate, actinomycin-D or etoposide AUC were not significantly predictive of efficacy or toxicity (*P*>0.1 throughout), nor was MTX C_SS_ at 12 h. 7-Hydroxymethotrexate AUC_0−*∞*_ was not predictive of renal toxicity, as indicated by a >30% increase in serum creatinine (931±518 *vs* 761±468 *μ*g ml^−1^h^−1^ in patients with and without renal toxicity, respectively, *P*>0.1).

As an overall measure of total cytotoxic exposure, the AUC values for each drug were normalised (AUC/mean AUC) and summed up. These values were also not predictive of efficacy (1.9±0.3 *vs* 2.0±0.7, *P*>0.1) or toxicity (1.9±0.7 *vs* 2.0±0.4, *P*>0.1) for MTX+etoposide or for MTX+etoposide +actinomycin-D (2.9±0.4 *vs* 3.0±1.2 and 2.8±1.1 *vs* 2.8±0.6, respectively, *P*>0.1). Grouping patients according to individual PK values being greater or less than the median values suggested an increased likelihood of treatment failure in patients with MTX AUC values <median (13/21 *vs* 7/22, *P*=0.048).

## DISCUSSION

We show that the novel regimen GAMEC, with appropriate surgery, produces a PFS of 74% in untreated patients and 51% in previously treated patients. These data suggest this treatment to be highly active in GCT. The permissive entry criteria allowing patients without formal histology to enter the study meant that very ill patients could be treated using this protocol. In addition, the use of an induction cycle of baby BOP allowed these patients to be stabilised before commencing intensive therapy, and the use of HD MTX here negated the requirement for cranial irradiation.

Many studies for intensive protocols in untreated GCT have employed large doses of bleomycin, making them unsuitable for salvage treatment. This regimen did not include bleomycin. Concerns that this might compromise outcome could be off-set by the fact that actinomycin-D produces a response rate of 38%, which is greater than that of bleomycin (Early and Albert, 1976).

The regimen was accompanied by significant toxicity, with five TRDs. These patients were older and one had only recently been exubated following a laparotomy, and was severely malnourished.

There was a high frequency of mucositis and febrile neutropenia. Many cycles required the use of platelets, particularly if the patients had been pretreated (Table 3b). It proved difficult to deliver the treatment in older patients (>35 years), and this was the probable reason for their inferior survival. Nevertheless, for those who relapsed, it was possible to give further therapy to all of the patients in the untreated group and to the vast majority (16/17) of the pretreated group. Adequate stem cell collections were also possible in 9/11 patients in whom it were attempted, suggesting that deferring high-dose chemotherapy for patients who relapsed after GAMEC was practical.

We have shown that it is possible to deliver HD MTX in combination with cisplatin (cisplatin given 38 h after HD MTX). The measurement of the serum creatinine 24 h post-MTX allowed identification of patients who had developed renal impairment, and future chemotherapy could be dosed accordingly. The results of the pharmacokinetic studies lead to the conclusion that 7-OH MTX is not the cause of MTX-induced renal dysfunction, as has been widely held to be the case. In these patients, cisplatin was withheld until the serum creatinine fell, with levels checked every 12 h. The role of MTX in relapsed disease remains controversial, while single-agent data have proved to be disappointing. However, regimens incorporating MTX have proved to be encouraging. Two other intensive regimens have used MTX in intermediate doses (BOMP/EPI (Germa-Lluch *et al*, 1999) and POMB/ACE (Husband and Green, 1992)). Both used alkylating agents, although the dose in POMB/ACE was low. The single-agent data in relapsed disease used 1 g m^−2^ (Atkinson *et al*, 1987), substantially lower than that used here.

The median delivery of cisplatin was 250 mg m^−2^ in the pretreated and 270 mg m^−2^ in the untreated patients, suggesting the targeted delivery of 300 mg m^−2^ to be realistic. The relationship between dose density and outcome could only be found in the pretreated group. It appeared that maintenance of dose density over the first two cycles was the most important factor, with dose intensity and absence of dose delay being equally important. The failure to see such a relationship in the untreated group probably reflects the low number of relapses in this group in the first place, and this is similar to the finding with POMB/ACE chemotherapy (Husband and Green, 1992). The failure of double-dose cisplatin in BEP to improve survival in untreated patients questions the wisdom of sole escalation of cisplatin as a major strategy to improve outcome (Nichols *et al*, 1991).

For relapsed patients, the data presented are comparable to that from other groups using high-dose therapy (Bhatia *et al*, 2000; Motzer *et al*, 2000). Other groups have reported encouraging results using alternative agents, for instance cisplatin and epirubicin (Bedano *et al*, 2005), and recently an interesting case report suggested that antiangiogenic agents may also have a role (Voigt *et al*, 2006).

We failed to detect a significant difference in the outcome of these patients, when the two most commonly used scoring systems for prognosis (MRC (Fossa *et al*, 1999) and MSKCC (McCaffrey *et al*, 1997)) were applied. It appears that the good group (using the MSKCC criteria), for whom it has been suggested that the cisplatin, ifosfamide and paclitaxel (TIP)-based approach is frequently curative, fare as well as those reported (67% with GAMEC *vs* 65%) (Kondagunta *et al*, 2005). Results from the poor-risk relapse group, for whom conventional therapy seems inadequate, appear as good as those reported for ifosfamide/paclitaxel induction followed by three cycles of high-dose carboplatin and etoposide treatment, supported by autologous blood stem cells (50% with GAMEC *vs* 41%) (Motzer *et al*, 2000).

We could only identify two prognostic factors that correlated with poor outcome, namely age and raised LDH before GAMEC. It might have been that these simply were the patients who received a lower intensity of therapy. In fact all of the six patients with raised LDH who received <80% dose density over the first two cycles, relapsed *vs* four of eight of those who received the higher dose intensity. A similar finding existed for age (2/10 *vs* 3/6). Although not statistically significant, there is a suggestion that adequate dose intensity may overcome these adverse prognostic factors.

Late relapses appear salvageable with GAMEC (5/9), a group thought to be chemorefractory. Recently, a similar finding has been described with TIP (7/14) (Ronnen *et al*, 2005). Derived PK values for MTX, actinomycin-D and etoposide clearance were inline with previously published values (Clark *et al*, 1994; Crews *et al*, 2004; Veal *et al*, 2005). These were not predictive of outcome. The use of multiple drugs was a confounding factor, especially as cisplatin pharmacokinetics were not assessed. The use of filgrastim and folinate will have reduced the impact of pharmacokinetic variability on myelosupression. Despite these caveats, there was a trend towards lower MTX AUC in patients who relapsed.

Overall, these results show that GAMEC is an effective therapy both for untreated patients and those who relapse. Further relapses can still receive treatment thereafter. The data suggest that the current prognostic scoring systems for relapsed patients fail to identify a poor-prognosis group for this regimen. These encouraging results have been obtained with established agents.

## Figures and Tables

**Table 1:** Patient characteristics

	**Pretreated patients**	**Untreated patients**
	**(35)**	**(27)**
Median age (range)	24.6 (17.8–53.2)	31.1 (16–42.6)

*Histology*		
Non-seminoma	26	17
Seminoma	7	0
Mixed	1	1
Unknown	1	9

*Initial IGCCCG*		
Good	20	N/A
Intermediate	8	N/A
Poor	7	27

*Orchidectomy*		
Yes	24	9
No	11	18

*Outcome to prev chemo*		
CR	8	—
M−ve PR	21	—
M+ve PR	4	—
PD	2	—

*No prev therapies*		
1	29	0
2	6	0

*HCG at start of GAMEC*		
Median	1693 (1–106 000)	112 559 (0–33 5000)

*AFP at start of GAME*		
Median	38 (3–11 352)	1427.5 (3–43 500)

*LDH at start of GAMEC*		
Median	480 (260–3880)	1123 (451–3661)
Raised	14 (median 1 × normal)	25 (median 2.3 × normal)

*Dx sites at start of GAMEC*		
Lung	21	24
LNs	27	23
Liver	3	9
CNS	4	5
Other	8 (see 2 below)	14 (see 3 below)

Abbreviations: AFP, alpha foetoprotein; CR, complete remission; HCG, human chorionic gonadotrophin; LDH, lactate dehydrogenase; M−ve PR, marker-negative partial response; M+ve PR, marker-positive partial response; N/A, not applicable.

Peritoneum, scrotum, testes, mediastinum, bone, inferior vena cava;

Renal, bone, spleen, adrenal, stomach, pericardium, testes, kidney, pleural effusion, skin, ascites, intestine, ivc.

**Table 2:** Outcome to GAMEC

	**Pretreated patients**		**Untreated patients**	
	35		27	
Median number cycles	4 (range 2–5)		5 (range 1–5)	
Baby BOP induction	2		10	
Response to GAMEC	Number	PF	Number	PF
CR	3	3	3	2
SCCR	10	10	12	12
SICR (good)	3	3	0	0
SICR (poor)	5	0	0	0
M−ve PR	6	2	9	6
M+ve PR	2	0	1	0
M−ve SD	1	0	0	0
SD	1	0	0	0
PD	1	0	0	0
TRD	3	0	2	0
Total PF to GAMEC+surgery		18 (51%)		20 (74%)
PF to other therapy		4 (11%)		1 (4%)
Total PF		22 (63%)		21 (78%)

Abbreviations: CR, complete remission; M−ve PR, normalisation of tumour markers with residual masses; M+ve PR, at least 90% reduction in markers for at least 1 month; PD, progressive disease; PF, progression free; SCCR, surgically confirmed CR; SD, stable disease; SICR, surgically induced CR (viable cancer found at surgery, but all sites of disease removed); TRD, treatment-related death.

<5% viable cancer=good; >5% viable cancer=poor.

**Table 3a:** Percentage of patients having grade 3/4 WHO toxicities

	**Pretreated**					**Untreated**				
**Cycle**	**1**	**2**	**3**	**4**	**5**	**1**	**2**	**3**	**4**	**5**
Number of patients	35	35	29	21	17	27	26	26	24	18
Alopoecia	35	91	96	100	95	27	96	100	100	100
Anorexia	6	18	7	5	11	12	16	21	24	31
Diarrhoea	9	6	11	0	5	12	0	4	5	8
Constipation	0	0	4	0	0	0	0	0	0	0
Infection	59	68	70	43	37	69	44	54	57	46
Lethargy	24	41	52	40	31	23	28	38	33	23
Mucositis	27	29	41	10	16	15	28	33	38	8
Nausea and vomiting	3	9	4	0	0	4	4	8	0	8
Neuropathy	0	6	0	15	11	0	0	0	0	0
Neutropenia	91	93	85	95	61	93	93	83	80	67
Thrombocytopenia	82	96	89	95	78	85	96	67	90	93

**Table 3b:** Blood product requirements

	**Pts requiring platelets**			**Pts requiring blood transfusion**	
	***N***	**%**	**Med.**	**%**	**Med.**
*Pretreated patients*					
Cycle 1	35	37	1	37	3
Cycle 2	35	54	2	66	3
Cycle 3	29	59	2	69	3
Cycle 4	21	43	3	71	3
*Cycle 5*	17	41	2	59	3

*Untreated patients*					
Cycle 1	27	22	3	44	6
Cycle 2	26	31	1.5	38	4.5
Cycle 3	26	50	1	58	3
Cycle 4	24	46	1`	62	3
Cycle 5	18	39	1	50	3

Abbreviation: Pts, patients.

**Table 4:** Pretreated patients: drug density and PF status to GAMEC

**Pretreated Pts: drug density and PF to GAMEC**			
	**Cycles 1–2**		
% dose density	<80%	⩾80%	*P* (FET)
Actinomycin	5/12 (42%)	13/22 (59%)	0.48
Methotrexate	1/9 (11%)	17/25 (68%)	0.006
Etoposide	4/12 (33%)	14/22 (64%)	0.15
Cisplatin	8 /18 (44%)	10/16 (62%)	0.33
Combined	3/14 (21%)	15/20 (75%)	0.004

Abbreviations: FET, Fisher's exact test; PF, progression free; Pts, patients.

**Table 5:** Pharmacokinetic data on MTX, 7-OH MTX, actinomycin-D and etoposide

	**AUC_0–∞_** (*μ***g ml^−1^ h^−1^**)	**CL (ml min^−1^ m^−2^)**	**Half-life (h)**
Methotrexate	2730±1444	72±40 (from AUC)	4.6±1.4
		94±58 (from C_SS_)	
7-OH methotrexate	855±439	—	13.2±6.0
Actinomycin-D	0.110±0.077	190±84	19.3±9.5
Etoposide	93±24	16.3±4.8	6.7±2.1

Abbreviation: AUC, area under the concentration–time curve; MTX, methotrexate.
